# Upregulation of calpain activity precedes tau phosphorylation and loss of synaptic proteins in Alzheimer’s disease brain

**DOI:** 10.1186/s40478-016-0299-2

**Published:** 2016-03-31

**Authors:** Ksenia Kurbatskaya, Emma C. Phillips, Cara L. Croft, Giacomo Dentoni, Martina M. Hughes, Matthew A. Wade, Safa Al-Sarraj, Claire Troakes, Michael J. O’Neill, Beatriz G. Perez-Nievas, Diane P. Hanger, Wendy Noble

**Affiliations:** Department of Basic and Clinical Neuroscience, Maurice Wohl Clinical Neuroscience Institute, King’s College London, Institute of Psychiatry, Psychology and Neuroscience, Rm1.25, 5 Cutcombe Road, Camberwell, London, SE5 9RX UK; King’s College London, MRC London Neurodegenerative Diseases Brain Bank, London, UK; Eli Lilly and Company, Erl Wood Manor, Windlesham, Surrey GU20 6PH UK

**Keywords:** Alzheimer’s disease, Calpain, GSK-3, Tau, Synapse, Braak stage, Postmortem brain

## Abstract

Alterations in calcium homeostasis are widely reported to contribute to synaptic degeneration and neuronal loss in Alzheimer’s disease. Elevated cytosolic calcium concentrations lead to activation of the calcium-sensitive cysteine protease, calpain, which has a number of substrates known to be abnormally regulated in disease. Analysis of human brain has shown that calpain activity is elevated in AD compared to controls, and that calpain-mediated proteolysis regulates the activity of important disease-associated proteins including the tau kinases cyclin-dependent kinase 5 and glycogen kinase synthase-3. Here, we sought to investigate the likely temporal association between these changes during the development of sporadic AD using Braak staged post-mortem brain. Quantification of protein amounts in these tissues showed increased activity of calpain-1 from Braak stage III onwards in comparison to controls, extending previous findings that calpain-1 is upregulated at end-stage disease, and suggesting that activation of calcium-sensitive signalling pathways are sustained from early stages of disease development. Increases in calpain-1 activity were associated with elevated activity of the endogenous calpain inhibitor, calpastatin, itself a known calpain substrate. Activation of the tau kinases, glycogen-kinase synthase-3 and cyclin-dependent kinase 5 were also found to occur in Braak stage II-III brain, and these preceded global elevations in tau phosphorylation and the loss of post-synaptic markers. In addition, we identified transient increases in total amyloid precursor protein and pre-synaptic markers in Braak stage II-III brain, that were lost by end stage Alzheimer's disease, that may be indicative of endogenous compensatory responses to the initial stages of neurodegeneration. These findings provide insight into the molecular events that underpin the progression of Alzheimer's disease, and further highlight the rationale for investigating novel treatment strategies that are based on preventing abnormal calcium homeostasis or blocking increases in the activity of calpain or important calpain substrates.

**Electronic supplementary material:**

The online version of this article (doi:10.1186/s40478-016-0299-2) contains supplementary material, which is available to authorized users.

## Introduction

Synaptic dysfunction and neurodegeneration in Alzheimer’s disease (AD) is associated with the presence of extracellular deposits of β-amyloid (Aβ) in neuritic plaques and intraneuronal neurofibrillary tangles containing abnormally phosphorylated and aggregated tau [50]. Considerable evidence has shown that disruptions to Ca^2+^ signalling pathways are associated with neuronal loss in AD [6]. Elevated Aβ burden leads to increased intracellular Ca^2+^ concentrations [36] by several mechanisms including increased Ca^2+^ entry through native ion channels and receptors [68, 76] or amyloid pores [15], release of Ca^2+^ from intracellular stores [18, 68] and inactivation of the ionic machinery that extrudes excess Ca^2+^ from neural cells [2]. Sustained increases in intracellular Ca^2+^ leads to activation of many calcium-sensitive proteins implicated in AD including calcium/calmodulin-dependent protein kinase (CAMKK2; [44]), calcineurin [46, 67, 77], and calpains [2, 71].

Calpains are a family of cysteine proteases closely linked with AD. They cleave amyloid precursor protein (APP) to regulate Aβ production [47], several synaptic proteins including dynamin-1 and the NMDA receptor subunit NR2B to affect synapse health [64], and the clathrin adapter protein PICALM to modulate endocytosis [1]. Much research has also highlighted the actions of calpain for disease-associated changes in tau. Calpain can cleave the N-terminus of tau directly to generate neurotoxic tau fragments [17, 32]. Calpain-mediated proteolysis of kinases or their activators regulates the activity of key tau kinases, such as GSK-3 [25] and cdk5 [40], both of which promote tau phosphorylation and tau-associated neurodegeneration in vivo [12, 23, 51, 52].

Evidence from the study of postmortem brain supports an important role for aberrant calpain regulation in AD. Calpain activity is increased in end-stage AD brain [2, 32, 61], particularly in neurofibrillary tangle-containing neurons [25], and elevated cleavage of many calpain substrates has been demonstrated in postmortem AD brain [2, 42, 43]. Indeed, a recent study using end-stage AD brain demonstrated a strong link between calpain activation, N-terminal cleavage and activation of GSK-3 and tau phosphorylation at several disease-relevant epitopes [32].

The aim of this study was to determine the temporal association between changes in calpain, tau kinases, tau and synaptic proteins during the development of sporadic AD using brain tissue from Braak stage II to VI AD and age-matched controls. We observed increased activity of calpain-1 from mid-stages of AD. These increases were associated with elevated activity of the tau kinases, GSK-3 and cdk5 in Braak stage II-III, which were in turn observed prior to elevated tau phosphorylation and loss of synaptic markers. These data extend previous findings that calpain-1 and tau kinases are upregulated at end-stage AD, and suggest that calcium-sensitive signalling pathways are activated very early during disease development, prior to changes in tau phosphorylation and synapse loss. These findings further highlight the rationale for investigating novel treatment strategies for AD that are based on preventing abnormal calcium homeostasis or blocking increases in calpain or tau kinase activities.

## Materials and methods

### Preparation of post mortem human brain lysates

Frozen postmortem human temporal cortex (Table 1) from control (*n =* 5) and pathologically confirmed cases of sporadic AD of Braak stage II (*n =* 5), III (3), IV (*n =* 4), V (*n =* 3) and VI (*n =* 5) were obtained from the MRC London Neurodegenerative Diseases Brain Bank (Additional file 1: Table S1, Additional file 2: Table S2). Frozen tissue was homogenized (0.5–1 mg mL^−1^) in ice-cold lysis buffer containing 50 mM Tris-buffered saline (TBS, pH 7.4), 0.1 % (v/v) Triton X-100, 10 mM sodium fluoride, 1 mM sodium orthovanadate, 2 mM ethylene glycol tetraacetic acid (EGTA), 1 mM phenylmethylsulfonyl fluoride (PMSF) and Complete™ protease inhibitor (Roche Diagnostics Ltd., West Sussex, UK). Homogenates were centrifuged at 25,000_g(av)_ for 20 min at 4 °C. The resulting supernatants were collected and stored at −20 °C until required. Protein concentrations in supernatants were measured using a BCA protein assay kit (Pierce Endogen, Rockford, USA). Samples were normalised to equal protein concentration before being analyzed by western blotting or ELISA. Pellets were resuspended in 4 x sample buffer containing 50 mM Tris–HCl pH 7.2, 2 % (w/v) SDS, 10 % (v/v) glycerol, 2.5 % (v/v) β-mercaptoethanol, 12.5 mM EDTA, 0.02 % (w/v) bromophenol blue, briefly sonicated, and heated to 95C for 5 min prior to western blotting.

### Isolation of sarkosyl insoluble tau

Sarkosyl extractions were performed as previously described by us [52]. Briefly, tissue was homogenized in 50 mM TBS (pH 7.4) containing 2 mM EGTA, 1 mM sodium orthovanadate, 10 mM sodium fluoride and 1 mM PMSF at 100 mg/mL (w/v), and centrifuged at 20 000 gav for 20 min at 4 °C. Sarkosyl (10 % v/v) was added to the resulting supernatant to give a final concentration of 1 % (v/v), and samples mixed for 30 min at ambient temperature with rocking and then centrifuged at 100,000 gav for 60 min at ambient temperature. The supernatant was collected and the pellet washed twice with 1 % sarkosyl, prior to solublization in 2 × SDS sample buffer. Thus, three fractions were generated, containing: (i) low speed supernatant (ii) sarkosyl-soluble and (iii) sarkosyl-insoluble tau. Samples were subjected to immunoblotting, standardising the amount of sarkosyl-soluble or -insoluble tau to the amount of tau present in low speed supernatants.

### Gel electrophoresis and Western blotting

Protein was electrophoresed on 10–12 % (w/v) SDS-polyacrylamide gels. Separated proteins were transferred to nitrocellulose membranes (Whatman, Maidstone, UK) and either blocked with 5 % (w/v) non-fat milk in TBS, 5 % (w/v) bovine serum albumin (BSA) in TBS or Odyssey® Blocking Buffer for 1 h. After blocking, membranes were incubated overnight at 4 °C in blocking solution containing appropriate dilutions of primary antibody. Blots were washed and incubated with fluorophore-conjugated secondary antibodies for 1 h at ambient temperature. Proteins were visualized using an Odyssey® Infrared Imaging system (Li-Cor Biosciences, Cambridge, UK) and quantified using ImageJ (NIH, Maryland, USA) or proprietary Odyssey sa software (Li-Cor Biosciences, Cambridge, UK).

Human postmortem brain samples were run on multiple gels, each containing a standard control to enable comparison of samples across gels. Statistical analysis was performed following standardization of total protein amounts against neuron-specific enolase (NSE) or β-actin amounts in each sample. White lines separating lanes in immunoblot images indicate splicing together of different blots or different regions of the same blot.

### Antibodies

The following primary antibodies were used for Western blotting: calpain-1 large active subunit (No. 28257, rabbit IgG; Abcam plc, Cambridge, UK), calpastatin (CAST, No. 4146, rabbit IgG; Cell Signalling Ltd, Beverly, MA, USA); cleaved (active) caspase-3 (No. 13847, Asp175/Ser376, rabbit IgG; Abcam plc, Cambridge, UK), spectrin, α chain (MAB1622, Clone AA6, mouse IgG; Merck KGaA, Darmstadt, Germany), total tau (DAKO, A0024, rabbit IgG; Agilent Technologies, Glostrup, Denmark), tau dephosphorylated at Ser199/202 (Tau-1, MAB3420, Clone PC1C6, mouse IgG; Merck KGaA, Darmstadt, Germany), tau phosphorylated at Ser202 (CP13, mouse IgG; P. Davies, Feinstein Institute for Medical Research, NY, USA), tau phosphorylated at Ser396/404 (PHF1, mouse IgG; P. Davies, Feinstein Institute for Medical Research, NY, USA), glycogen synthase kinase-3α/β phosphorylated at Ser21/9 (pGSK3, No. 9331, rabbit IgG; Cell Signalling Ltd, Beverly, MA, USA), total GSK3α/β (GSK3, SA364-0100, Clone 1H8, mouse IgG; Enzo Life Sciences Inc, Exeter, UK), cdk5 (sc-6247, Clone J-3, mouse IgG, Santa Cruz Biotechnology, Dallas, Texas, USA), p35 (sc-820, Clone C-19, rabbit IgG, Santa Cruz Biotechnology, Dallas, Texas, USA), β-amyloid, 1–16 (6E10, SIG-39300, mouse IgG; Covance, California, USA), NR2B (No. 06–600, rabbit IgG; Merck KGaA, Darmstadt, Germany), PSD-95 (No. 2507, rabbit IgG; Cell Signalling Ltd, Beverly, MA, USA), synapsin I (AB1543P, rabbit IgG, Merck KGaA, Darmstadt, Germany) and NSE (BBS/NC/VI-H14, mouse IgF; DAKO, Glostrup, Denmark). For immunohistochemistry antibodies against phosphorylated tau (clone [AT-8]; Autogen Bioclear UK Ltd, Wiltshire, UK) and amyloid β (Aβ) (Chemicon, Temecula, CA, USA) were used.

### Aβ1-40 and Aβ1-42 ELISA

Aβ1-40 and Aβ1-42 amounts in human brain samples were quantified using ELISA kits from Life Technologies, Paisley, UK (Aβ40 ELISA KHB3481; Aβ42 ELISA KHB3442) as previously described [73].

### Immunohistochemistry

As part of the neuropathological diagnosis of each case, 7 μm tissue sections were cut from formalin-fixed paraffin-embedded blocks of AD or control human brain tissue. Sections were deparaffinized and endogenous peroxidase activity was inhibited by incubating samples in 3 % (v/v) hydrogen peroxide for 30 min (for Aβ 80 % formic acid pretreatment for 1 h was used), and antigen retrieval was enhanced by microwaving in 10 mM sodium citrate buffer, pH 6.0. Sections were blocked for 20 min in 10 % normal serum before incubating with tau/Aβ antibodies overnight at 4 °C. Sections were then incubated with biotinylated secondary antibodies (DAKO) for 45 min. Sections were developed using the VECTASTAIN Elite ABC kit (Vector Laboratories) and 0.5 mg/ml 3,3′-diaminobenzidine chromogen (Sigma-Aldrich). All samples were counterstained with hematoxylin.

### Statistical analysis

Statistical analysis was performed using GraphPad Prism v6.0 (La Jolla, CA, USA). Western blot and ELISA data was analyzed by nonparametric one-way analysis of variance followed by Tukey’s post-hoc tests. Correlation analysis was performed using two-tailed Spearman tests with linear regression. Differences were considered statistically significant when *p <* 0.05. GraphPad Prism v6.0 was used for all statistical analyses.

## Results

Temporal cortex was used for these analyses, in keeping with previous studies of AD development [8]. Tau pathology, as assessed using immunohistochemistry, is minimal in the temporal cortex in the earliest stages of disease [8]. Therefore, examination of this region allowed us to determine changes in other proteins that precede pathological changes in tau in AD. In all cases, Braak stage II-VI tissues were compared with age-matched control brain, these latter tissues showing no evidence of neurodegeneration.

### Progressive accumulation of phosphorylated tau in AD

Tau is a microtubule-binding protein that is abnormally phosphorylated and progressively accumulates in NFTs in AD [26]. Abnormal processing of tau is closely linked with synaptic and neuronal dysfunction in AD [11], and is an increasingly important target for the development of new dementia therapies [53].

**Fig. 1:**
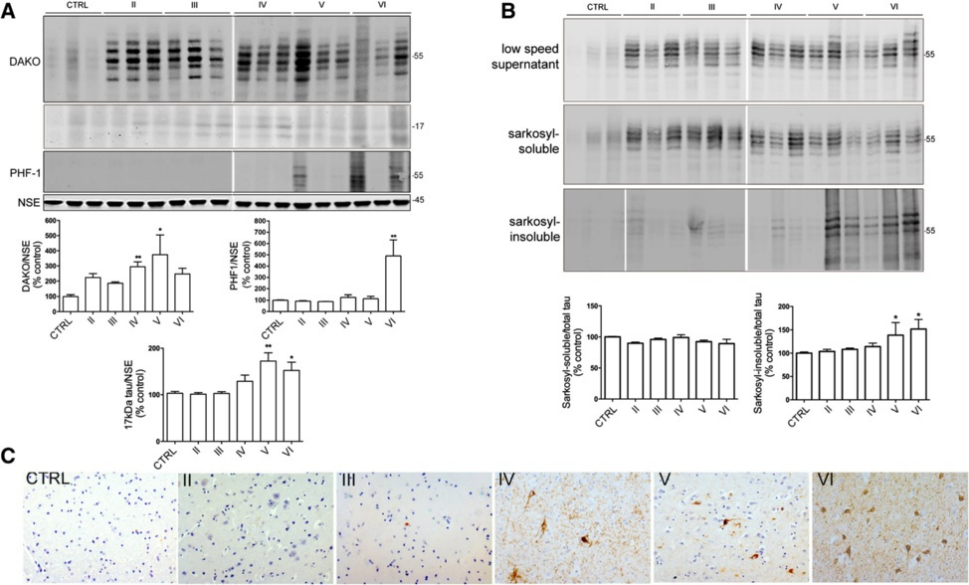
Total tau amounts are elevated throughout AD progression, whereas increased tau phosphorylation is only detectable at end-stage disease. **a** Representative immunoblots of cortical homogenates from postmortem brain. Blots were probed with antibodies to detect total (phosphorylated and non-phosphorylated) amounts of tau (DAKO) at 50 to 70 kDa, and tau phosphorylated at Ser396/404 (PHF-1) at 50 to 70 kDa. Blots were also probed with an antibody against neuron-specific enolase (NSE, 45 kDa) which acted as a loading control. Bar graphs show the amounts of DAKO and PHF-1 once standardized to NSE content in each sample. **b** Representative immunoblots of samples from sarkosyl extraction protocols showing low speed supernatants, sarkosyl-soluble and sarkosyl-insoluble tau probed with antibodies against total tau (DAKO). Bar charts show sarkosyl-soluble and sarkosyl-insoluble tau as a proportion of tau in low speed supernatants as a measure of total tau. **c** Postmortem brain sections immunostained with an anti-tau (AT8) antibody show Braak staging of AD brain. NFTs are absent from age-matched control brain. CTRL: control (*n =* 5), Braak II AD (*n =* 4), Braak III AD (*n =* 3), Braak IV AD (*n =* 4), Braak V AD (*n =* 3), Braak VI AD (*n =* 5). Data is mean ± SEM. *p < 0.05, **p < 0.01

Postmortem brain lysates were immunoblotted using antibodies against total tau (DAKO) and tau phosphorylated at Ser396/404 (PHF1), both of which yielded bands of the expected size, approximately 50–68 kDa (Fig. 1a). Bands of approximately 17 kDa were also detected, which may correspond to the 17 kDa calpain-cleaved tau fragments previously described by others [17, 32]. Blots were also probed with an antibody against NSE, which acted as a control for gliosis and/or loss of protein during neuron loss and postmortem delay. Total tau protein is reported to be increased in degenerating regions of AD brain [35]. Following normalization of tau amounts to NSE, we found an increase in total tau protein in mid-late stage AD. Tau protein amounts were significantly increased in Braak stage IV and V tissues compared to control (*p <* 0.05), with the lack of significance at Braak stage II, III and VI likely to reflect the relatively small sample set used in this study since clear elevations in tau amounts can be observed in these samples by western blotting (Fig. 1a). Quantification of tau phosphorylated at Ser396/404, as detected by the PHF-1 antibody, showed that tau phosphorylation at this epitope is below the detectable range in control brain, and in most Braak stage II-V tissues, but was significantly increased at end-stage AD (Braak VI) when compared to control (*p <* 0.001; Fig. 1a). Similar findings were observed when these samples were blotted with an antibody against tau phosphorylated at Ser202 (CP13, data not shown).

To determine if the increased abundance of tau in these samples results from the accumulation of degradation-resistant tau aggregates, insoluble tau was isolated from postmortem brains with sarkosyl. This protocol results in three tau fractions, a low speed supernatant (S1), sarkosyl-soluble (S2) and sarkosyl-insoluble tau (P2), all of which were immunoblotted with antibodies against total tau and pSer396/404 (PHF1). These findings confirmed an increase in insoluble tau as a proportion of total tau in Braak stage V and VI tissues relative to earlier Braak stages and controls (Fig. 1b). Thus, the increase in total tau protein observed in Fig. 1a likely reflects the accumulation of this insoluble tau in tissue lysates, particularly since no changes in total tau mRNA have been reported in sporadic AD cortex [7, 28].

Immunohistochemical studies of fixed postmortem cortex labelled with the AT8 phospho-antibody are shown to confirm the Braak staging of these samples; these show progressive appearance of characteristic tangle-like structures in Braak IV-VI tissues (Fig. 1c).

### Total APP amounts are increased in Braak stage II-III brain

**Fig. 2:**
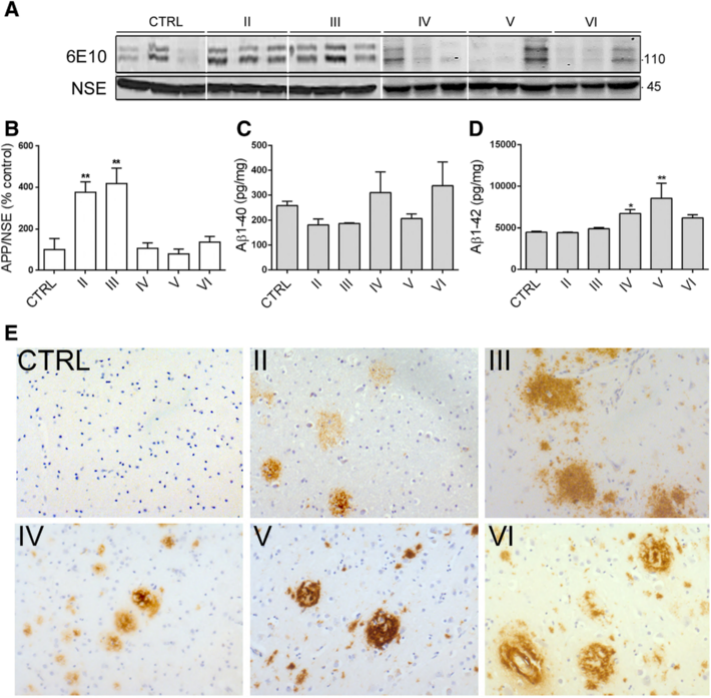
Transient elevations of total APP amounts in early AD, and persistent accumulation of Aβ1-42 at end-stage disease. **a** Representative immunoblots of cortical homogenates from postmortem brain. Blots were probed with the 6E10 antibody to detect full-length amyloid precursor protein (APP) at 110 to 130 kDa. Blots were also probed with an anti-neuron-specific enolase (NSE, 45 kDa) as a loading control. **b** Bar graph shows APP amounts in brain following standardization to NSE protein in the same sample. Aβ ELISAs were used to measure Aβ1-40 and Aβ1-42 amounts in pg mg^−1^ in these tissues. Bar graphs show (**c**) Aβ1-40 and (**d**) Aβ1-42 amounts in each sample. **e** postmortem brain sections immunostained with an anti-Aβ antibody show the progressive development of amyloid plaque pathology in AD brain. CTRL: control (*n =* 5), Braak II AD (*n =* 4), Braak III AD (*n =* 3), Braak IV AD (*n =* 4), Braak V AD (*n =* 3), Braak VI AD (*n =* 5). Data is mean ± SEM. *p < 0.05, **p < 0.01

Amyloid precursor protein (APP) is a type 1 transmembrane glycoprotein that, in AD, is pathologically cleaved to give rise to Aβ peptides of varying length [13]. We assessed amounts of APP holoprotein in postmortem cortex by probing blots with an antibody specific for C-terminal APP (6E10), which yielded two main bands at 106 and 113 kDa and a faint band at 130 kDa in late-stage AD brain, together characteristic of the three major APP isoforms found in human brain [14, 54], Fig. 2a). When standardized to NSE, total APP amounts were significantly increased in Braak stage II and III (*p <* 0.005) tissue compared to control, before returning to approximately control amounts in late (Braak IV-VI) stage AD (Fig. 2b). This finding extends previous studies which have shown no differences in total APP holoprotein amounts between control brain, brain from non-demented aged individuals and those with end-stage AD [54], by suggesting that there is an upregulation of APP when the first neurodegenerative changes occur in brain (Braak stage II-III), which possibly represents a compensatory CNS response to the first signs of damage in AD.

It would also have been of interest to examine the abundance of APP C-terminal fragments in these tissues. Although we have previous experience in blotting APP fragments [73, 74], it was very difficult to detect these small protein fragments in these tissues presumably due to rapid postmortem degradation (data not shown).

However, we measured total amounts of Aβ1-40 and Aβ1-42 in postmortem control and AD brain using specific Invitrogen ELISAs, as we have previously described [73, 74]. These analyses revealed that Aβ1-40 amounts did not significant differ between any stage of AD and control brain (Fig. 2c), whereas Aβ1-42 burden was significantly increased in the later stages of AD, showing significant increases at Braak stages IV (*p <* 0.05) and V (*p <* 0.001) when compared to controls (Fig. 2d). Elevated Aβ1-42 amounts have previously been demonstrated in cortical regions of sporadic AD brain [63]. Representative labelling of fixed sections with an antibody that detects Aβ is shown to confirm the presence of diffuse amyloid plaques in Braak II-III, and the appearance of dense core senile plaques in Braak stages IV-VI sections, none of which were found in control tissue (Fig. 2e).

The data presented here shows that marked increases in APP amounts are found transiently in Braak stage II-III stage AD brain, a change that might reflect an, as yet unknown, compensatory response to early stages of damage in the nervous system. These changes in APP preceded the elevated Aβ1-42 production and significant plaque deposition that was found in stage IV-VI AD brain.

### Calpain-1 activity is increased in Braak stage III brain and is sustained throughout disease progression

**Fig. 3:**
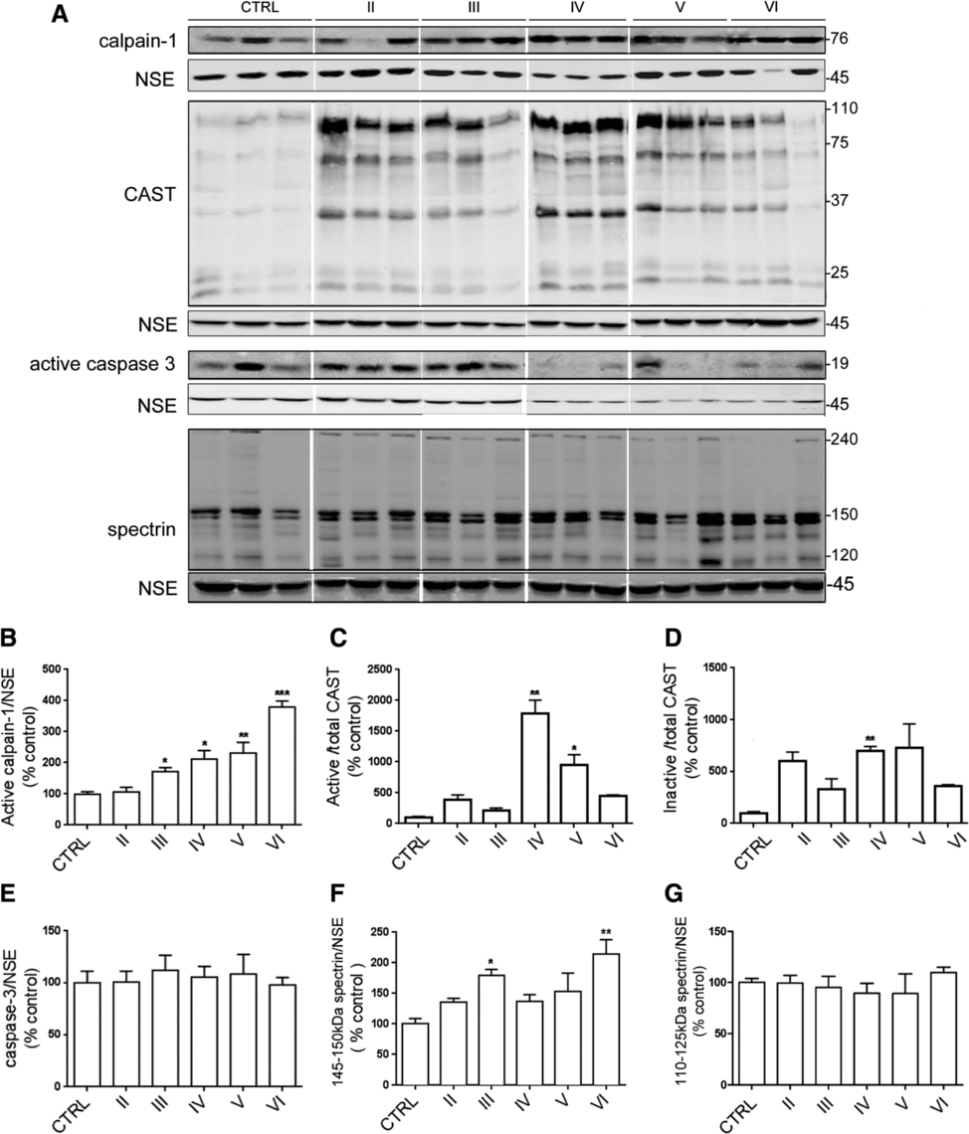
Active calpain-1 amounts are elevated early in AD and are sustained throughout disease progression. **a** Representative blots of cortical homogenates from postmortem brain. Blots were probed with antibodies to detect active calpain-1 at 76 kDa and active/cleaved caspase-3 at 19 kDa. An antibody against calpastatin (CAST) was used to detect CAST holoprotein at 110 kDa, active CAST at > 25 kDa and inactive CAST at < 25 kDa. An antibody against α-spectrin was used to detect holoprotein at 240 kDa, calpain- and caspase-cleaved fragments at 140 to 150 kDa and caspase-cleaved fragments at 110 to 130 kDa. Blots were also probed with an antibody against neuron-specific enolase (NSE, 45 kDa) as a loading control. Bar graphs show amounts of (**b**) active calpain-1 relative to NSE in each sample, (**c**) active CAST and (**d**) inactive CAST both as a proportion of total CAST (**e**) active caspase-3 relative to NSE, (**f**) caspase- and calpain- cleaved 140–150 kDa α-spectrin fragments and (**g**) caspase-cleaved 110–125 kD α-spectrin fragments, both standardized to NSE. CTRL: control (*n =* 5), Braak II AD (*n =* 4), Braak III AD (*n =* 3), Braak IV AD (*n =* 4), Braak V AD (*n =* 3), Braak VI AD (*n =* 5). Data is mean ± SEM. *p < 0.05, **p < 0.01, ***p < 0.001

Calpain-1 exists as an 80 kDa pro-enzyme that undergoes autolysis to yield 76 and 58 kDa active fragments [4, 75]. Blots of postmortem human brain were probed with an antibody that specifically detects the 76 kDa active calpain-1 subunit [2], revealing a single prominent band (Fig. 3a). The amounts of active calpain-1 were significantly increased in Braak stage III to VI tissues when compared to controls (Fig. 3b), indicating that elevations in calpain activity are prolonged from early-mid stages of AD.

### Calpastatin activity is increased at Braak stage IV to V, but this is not sustained at end-stage disease

Calpastatin (CAST) is the endogenous inhibitor of calpain in the brain, and thus plays an important role in responding to prolonged elevation of calpain [48]. Indeed, calpain is known to cleave CAST to generate active fragments with calpain inhibitory activity [25, 60]. We have previously shown that calpastatin activity is suppressed in late stage AD when compared to age-matched control brain [2]. To examine CAST activity throughout the development of AD, we probed blots of brain lysates with an antibody against CAST which detects CAST holoprotein at 110 kDa, a number of calpain-cleaved active CAST fragments at 37–75 kDa (which together with CAST holoprotein inhibit calpain) and bands below 37 kDa representing inactive CAST. The smaller CAST fragments are generated by caspase-1- and caspase-3-mediated cleavage of CAST and lack inhibitory activity [25, 60] (Fig. 3a). Active and inactive CAST were quantified separately as a proportion of total CAST (holoprotein plus all fragments). We found that levels of active CAST (holoprotein plus 37–75 kDa fragments) were significantly increased in Braak IV-V AD tissue (*p <* 0.05) compared to control (Fig. 3c). There were also differences, some significant, in the amounts of inactive CAST relative to total CAST in all AD tissues (Fig. 3d), likely representing the increased total CAST apparent in AD brain that was detected by immunoblotting.

### Active caspase-3 amounts do not change throughout AD progression

There is much evidence of crosstalk between calpains and caspases in the brain [48, 49], and both apoptotic and non-apoptotic activation of caspase-3 in discrete neurons has been demonstrated in AD brain [10, 59], although the pathological relevance of this is not clear [29]. Caspase-3 exists as a 32 kDa pro-enzyme which has limited catalytic activity, and as active fragments of 17- and 19-kDa that are generated by the action of caspase-8 and caspase-9, respectively. Here, blots of brain lysates were probed with an antibody against caspase-3 that detects both the pro-caspase and active fragments. As we found previously [2], this antibody detected predominantly a 19 kDa active caspase-3 band in postmortem brain (Fig. 3a). When the amounts of this active caspase-3 band were standardized to NSE amounts in the same sample, we found no significant increase in active caspase-3 in any AD group when compared to controls (Fig. 3e). This finding is in keeping with previous results from our group [2] and others [30].

### Cleavage of α-spectrin increases during AD progression

We next examined cleavage of the cytoskeletal protein α-spectrin as a surrogate marker of calpain-1 and caspase-3 activities. Blots were probed with an antibody against α-spectrin which detects bands of 240 kDa (holoprotein), calpain- and caspase-cleaved fragments (145 to 150 kDa) and caspase-3-cleaved fragments (110 to 130 kDa products) (Fig. 3a). Calpain- and caspase-cleaved α-spectrin bands were separately quantified following their normalization to NSE to control for any differences in protein loading. This quantification showed a general trend of increased levels of 145–150 kDa calpain and caspase-cleaved α-spectrin fragments from Braak stage II to VI, which was significantly different from control in Braak stage III (*p <*0.05) and VI (*p <* 0.001) brain (Fig. 3f). No differences were found in the amounts of caspase-cleaved α-spectrin fragments between any AD tissue and control. This suggests that the increased amounts of 145–150 kDa α-spectrin bands detected in AD are due to the action of calpain and not caspases, which is in keeping with our analysis of these protease activities (Fig. 3a–e).

### Cdk5/p25 is elevated in Braak III brains and is sustained to late-stage disease

**Fig. 4:**
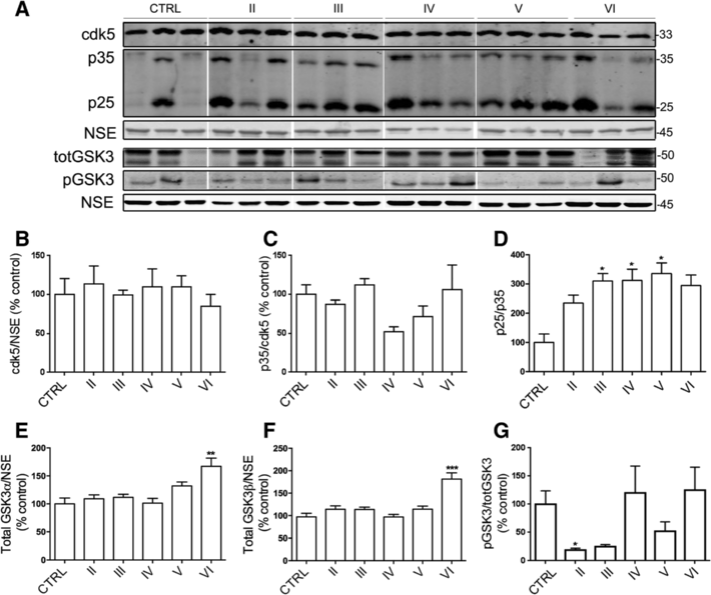
Changes in cdk5 and GSK-3 activities with AD progression. **a** Representative blots of cortical homogenates from postmortem brain. Blots were probed with antibodies against cyclin dependent kinase 5 (cdk5) to detect holoprotein at 33 kDa, p35 to detect holoprotein at 35 kDa and calpain-cleaved 25 kDa fragments (p25) at 25 kDa, total glycogen synthase kinase 3α/β (totGSK3) at 47 and 51 kDa, respectively and GSK3α/β phosphorylated at Ser21/9 (pGSK3). Blots were also probed with an antibody against neuron-specific enolase (NSE, 45 kDa) as a loading control. Bar graphs show amounts of (**b**) cdk5 relative to NSE, (**c**) p35 following normalisation to cdk5,  (**d**) the p25/p35 ratio, (**e**) GSK3α and (**f**) GSK3β relative to NSE, and (**g**) phosphorylated (inactive) GSK3 normalised to total GSK-3 protein. CTRL: control (*n =* 5), Braak II AD (*n =* 4), Braak III AD (*n =* 3), Braak IV AD (*n =* 4), Braak V AD (*n =* 3), Braak VI AD (*n =* 5). Data is mean ± SEM. *p < 0.05, **p < 0.01, ***p < 0.001

Cyclin-dependent kinase 5 (cdk5) is a proline-directed serine/threonine kinase that is somewhat controversially implicated in AD pathogenesis [19, 55, 69]. Cdk5 is activated when it forms a complex with one of its neuronal activators, such as p35. When cleaved by calpain, p35 yields the more stable and potent activator, p25, sustained expression of which is associated with increased tau phosphorylation and tau-associated synaptic and neuronal loss in vivo [12, 52]. Here, blots of AD brain lysates were probed with antibodies against cdk5, yielding a band of 33 kDa, and p35 which detects both p35 (35 kDa) and p25 (25 kDa) (Fig. 4a). Quantification of these results showed no significant changes in total cdk5 protein, p35 or p25 amounts in AD brain when compared to control (Fig. 4b, c). However, when p25 was measured as a proportion of p35, we found a significant increase in the p25/p35 ratio in Braak stage III to V brain (*p <* 0.05 for all) compared to control (Fig. 4d), the same disease stages in which calpain activity was found to be significantly elevated. This was indicative that increased calpain-mediated p25 generation and therefore increased cdk5 activity occurs from an early stage of AD development and is sustained throughout disease progression. It is worth noting that p25 levels are also reported to be decreased in AD [reviewed in 20], with increased p25 linked to synaptogenesis [20]. While the reasons for the differences between these studies and ours are not clear, it is possible that the elevated p25 amounts that we observe may be associated with the increased abundance of synaptic proteins, at least in early Braak stages.

### GSK3 expression and activity are increased in late stage AD brain

Glycogen synthase kinase 3 (GSK3) is a proline-directed serine/threonine kinase that plays a central role in AD pathology [26]. GSK-3 exists as two isoforms, GSK3α and GSK3β, which are phosphorylated at Ser21 and Ser9, respectively, to suppress kinase activity. GSK3 can be activated by calpain-mediated cleavage of the N-terminal portion of the kinase which removes Ser21/9 to allow an active kinase conformation [24]. Blots of brain lysates were probed with an antibody against GSK3α/β, yielding two bands at 51 and 47 kDa, which represent GSK3α and GSK3β, respectively (Fig. 4a). When normalized to NSE amounts in each sample, we found significantly increased levels of GSK3α (*p <* 0.001) and GSK3β (*p <* 0.0001) in Braak VI AD tissue compared to control (Fig. 4e, f). Blots were also probed with an antibody specific for GSK3α and GSK3β phosphorylated at Ser21/9 (pGSK3). We detected one prominent band in these tissues (Fig. 4a), which is likely to represent pSer9 on GSK-3β since the antibody we used exhibits preference for this site. We therefore did not differentiate between -α and –β isomers in our quantitative analysis. Our results indicated that phosphorylation of GSK3 is significantly reduced in Braak stage II AD brain when compared to control (Fig. 4g), indicating increased GSK3 activity at the very earliest stages of AD development. GSK-3 activity was not sustained throughout disease and was rather variable in later disease stages.

### Pre- and post-synaptic proteins are upregulated at Braak stage II-III and are lost in late-stage AD brain

**Fig. 5:**
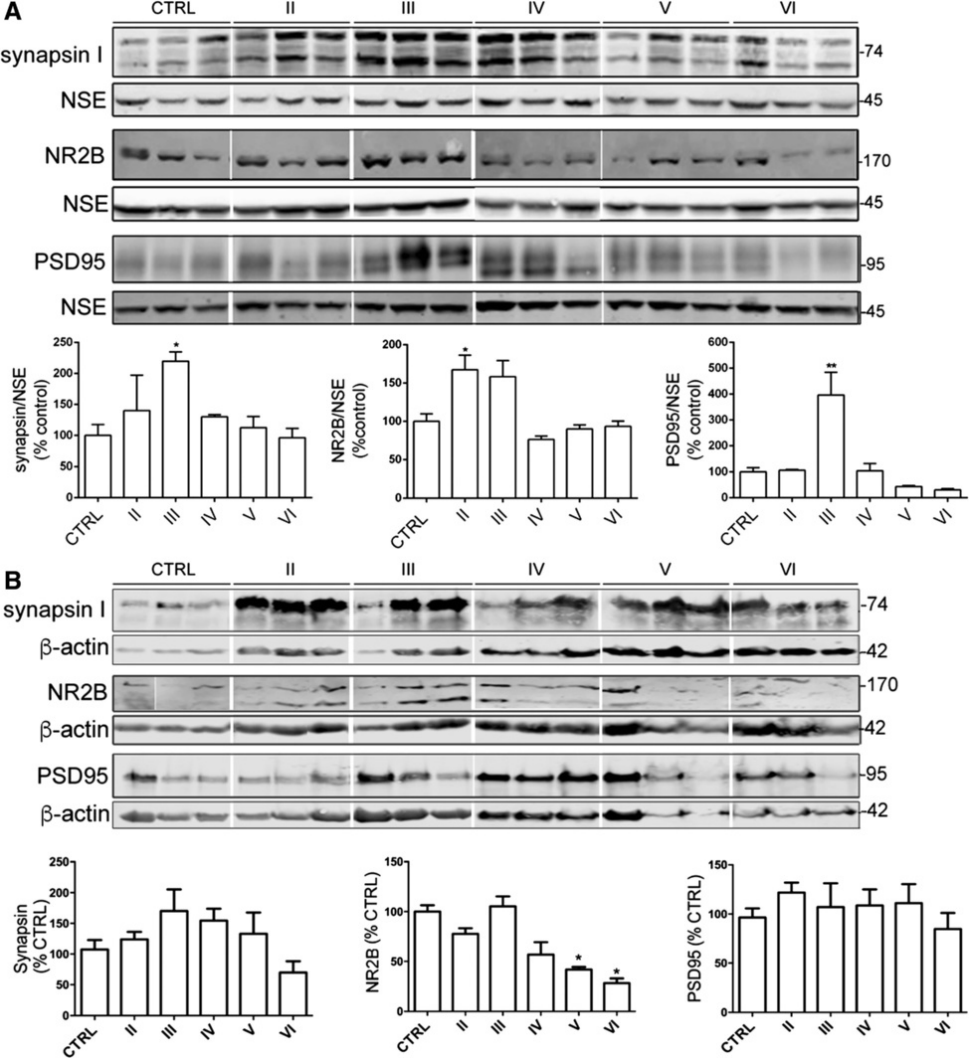
Pre- and post- synaptic protein amounts are altered during AD development. Representative blots of (**a**) supernatants and (**b**) pellets from cortical homogenates of postmortem brain. Blots were probed with antibodies against synapsin I holoprotein (74 kDa), the NR2B subunit of N-methyl D-aspartate receptor (NR2B,170 kDa) and post-synaptic density 95 protein (PSD95, 95 kDa. Blots were also probed with antibodies against neuron-specific enolase (NSE, 45 kDa) or β-actin (42 kDa) as loading controls. Bar graphs show amounts of synapsin I, NR2B and PSD95, all normalised to their respective loading control. CTRL: control (*n =* 5), Braak II AD (*n =* 4), Braak III AD (*n =* 3), Braak IV AD (*n =* 4), Braak V AD (*n =* 3), Braak VI AD (*n =* 5). Data is mean ± SEM. *p < 0.05, **p < 0.01

Alterations in intracellular Ca^2+^ and calpain activities, as well as the accumulation of phosphorylated tau, are linked with disrupted synaptic function in AD [11, 78]. We therefore assessed changes in synaptic markers in postmortem brain lysates. Blots were probed with an antibody against the pre-synaptic protein synapsin I, a neuron specific phosphoprotein localized to the cytoplasmic side of small synaptic vesicles that plays an important role in the release of neurotransmitters [3], which yielded two bands at approximately 70 and 74 kDa (Fig. 5a). To assess post-synaptic changes, antibodies against the NR2B subunit of N-methyl-D-aspartate (NMDA) receptors (170 kDa) and postsynaptic density-95 (PSD95, 95 kDa) were used (Fig. 5a). NR2B is a post-synaptic ionotropic glutamate receptor that conducts Ca^2+^ and mediates excitotoxic cell death in models of AD [29]. PSD-95 is an integral scaffolding component of the postsynapse that is also commonly used a marker for loss of synapses in AD models (e.g. [16]). All synaptic protein levels were normalized against NSE prior to statistical analysis.

Quantitative analysis revealed a similar pattern for all markers studied in the supernatant fraction, with increased protein levels apparent in Braak stages II-III relative to controls, followed by a recovery to normal levels or loss at end-stage disease. Synapsin-I and NR2B protein amounts were significantly increased in Braak stage II-III tissues, but were not different from control amounts in later stage AD (Fig. 5a). PSD95 protein amounts were also significantly increased in Braak III brains (*p <* 0.05) and were reduced below control amounts at end-stage AD (Braak stage VI) (Fig. 5a). These results perhaps suggest an increase in synapse number or activity during the early stages of AD, concomitant with increased APP protein amounts, which is lost as disease progresses and synapses degenerate.

In case the preparation of these samples resulted in synaptic proteins being lost in the pellets deposited by centrifugation, we also solubilized the respective protein pellets and immunoblotted these samples as described above. β-actin was used to normalize these blots so that the influence of neuron loss could be taken into account. In general, these blots showed a similar pattern to that observed when probing supernatants (Fig. 5b), with the exception of PSD-95 amounts which were much more stable across Braak stages. It is possible that this relates to the observation that PSD-95 is present in both cytoplasmic and postsynaptic membrane compartments [27]; the results here may suggest that there is tighter regulation of membrane-associated PSD-95 in disease.

We also detected NR2B fragments of approximately 150 kDa (Fig. 5b) in pellet fractions. These degradation products have previously been reported as an important measure of synaptic integrity [5]. Their presence indicates that there has been degradation of synaptic proteins in the tissues analysed here. However, we observed a direct correlation between the amounts of NR2B degradation products and full-length protein (*r =* 0.5017, *p =* 0.0339) therefore this degradation is believed not contributed to the changes in protein amounts reported here with respect to Braak stage.

### Calpain-1 correlates with Aβ1-42 burden, tau accumulation and tau kinase activity

**Fig. 6:**
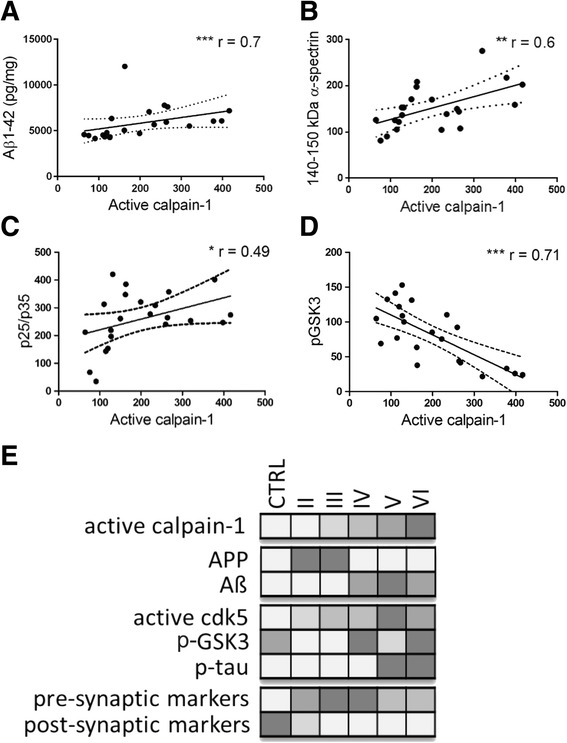
Calpain-1 activities in AD brain correlate with Aβ1-42 burden, cytoskeletal protein cleavage and kinase activities. Scatter plots show the correlation between amounts of active calpain-1 and (**a**) Aβ1-42, (**b**) calpain- and caspase-cleaved α-spectrin fragments and (**c**) p25 in all tissue samples. Correlation analysis was used to generate correlation co-efficients (r values) and significance. *p < 0.05, **p < 0.01, ***p < 0.001. **d** Qualitative plot illustrating the stage of disease at which changes were observed in calpain-1 activity, total APP protein, Aβ1-42 amounts, active cdk5 (p25/cdk5), active GSK-3 (reductions in pGSK-3), p-tau (tau phosphorylated at Ser396/404), pre- (synapsin-1) and post- (PSD-95) synaptic marker amounts. Relative amounts are indicated in grey scale, with low protein amounts signified by pale shading and large amounts by dark shading

Previous studies have linked elevated intracellular Ca^2+^ to Aβ overproduction, tau phosphorylation and synaptic dysfunction in AD [38, 83]. We therefore sought to determine whether elevated calpain-1 activity in postmortem brain of different AD stages correlated with other pathological findings, including tau phosphorylation and amounts, tau kinase activity, Aβ burden, and synaptic protein expression (Fig. 6).

We found that increased calpain-1 activity significantly correlated with increased total amounts of Aβ1-42 (*p <* 0.001; Fig. 6a), indicating that calpain may regulate APP processing or be activated by Aβ in AD brain, both of which mechanisms have previously been reported in vitro [45, 71]. Correlation of calpain-1 with calpain-cleaved α-spectrin and p25 amounts provided further confirmation of aberrant calpain-1 proteolytic activity in AD brain (*p <* 0.05 for both; Fig. 6b, c). Correlations between other proteins examined in this work did not yield any positive results (data not shown).

In summary, our findings demonstrate that the activity of the calcium-regulated protease, calpain, is elevated at early Braak stages (II-III), occurring alongside activation of the tau kinases cdk5 and GSK-3, and preceding accumulation of Aβ1-42, increases in phosphorylation of tau at disease relevant epitopes, and loss of synaptic markers at end-stage AD (Fig. 6d). In addition, we show increases in the amounts of APP, pre- and post-synaptic markers in Braak stage II-III AD brain that may represent some, as yet, unknown response of the nervous system to counteract the influence of early neurodegenerative changes.

## Discussion

Here we have used postmortem brain from all Braak stages to examine at which stage of disease development changes occur in key neurodegenerative disease proteins. We demonstrate that there is increased activity of calpain-1 from Braak stage III onwards in comparison to controls, extending previous findings that calpain-1 is upregulated at end-stage disease. In addition, activation of the tau kinases, GSK-3 and cdk5 were also found to occur in Braak stage II-III tissues, and these preceded global elevations in tau phosphorylation and the loss of post-synaptic markers observed in late-stage AD. In addition, we identified transient increases in total APP and pre-synaptic markers in Braak stage II-III, that were lost by end-stage AD, that may be indicative of endogenous compensatory responses to the initial stages of neurodegeneration. Our human brain data substantiate findings from many experimental models which have supported the hypothesis that sporadic AD arises in response to Aβ-mediated dysregulation of calcium signalling [6, 34, 37, 65, 66, 70].

Activation of calpain-1 was used as a marker for calcium dysregulation in this study. Calpain-1 is an intracellular cysteine protease that is activated upon autoproteolytic cleavage of the inactive precursor at its N-terminus in low micromolar (μM) concentrations of calcium. Increased truncation and activation of calpain-1 has previously been reported in late stage (Braak V-VI) AD brain [2, 25, 32, 61]. In addition, biomarker studies have recently demonstrated increased calpain activity in cerebrospinal fluid, and corresponding reductions of calpain activity in serum and plasma, in AD patients relative to non-cognitively impaired controls [39]. This is not surprising since calpain-mediated proteolysis has been implicated in many neurodegenerative pathways including the processing of amyloid precursor protein to generate Aβ species and resulting synaptic dysfunction [45, 72], cleavage and phosphorylation of tau by cdk5, GSK-3 and dual specificity tyrosine-phosphorylation regulated kinase 1A DYRK1A [25, 32, 50, 71], and altered learning and memory abilities via processing of synaptic proteins and suppression of LTP [33, 41].

In addition, recent evidence has implicated the calpain substrate and endogenous inhibitor, calpastatin in a novel autodestruction pathway linked to neurodegeneration [80, 81]. Rapid Wallerian degeneration of injured axons was shown to occur following activation of calpain alongside depletion of calpastatin inhibitory activity [31]. Induction of this calpain-calpastatin-mediated degeneration pathway was subsequently shown to occur downstream of nicotinamide mononucleotide adenylyltransferase 1-mediated changes in Sarm1 and mitogen activated protein kinase activities, and depletion of ATP [21, 32]. In addition to playing an important role in pruning processes during neuronal development [31], this pathway is likely to be involved in a wide spectrum of neurodegenerative diseases. Subsequent investigations will likely provide more insight into the importance of this signalling cascade for AD.

Another area in which dysregulation of calcium and/or calpain signalling is likely to be an important influence is the prion-like propagation of protein aggregates, a topic of intensive research in neurodegenerative disease research. Both Aβ and tau aggregates are reported to be transmitted through AD brain along anatomically connected pathways [82]. Although all of the mechanisms underlying pathology spread are not completely understood, stimulating electrical activity, or activating calcium-dependent NMDA and AMPA receptors, was shown to induce the release of tau from neurons in primary culture and in mouse models of disease [9, 58, 79]. Thus, it is possible that dysregulation of calcium-calpain pathways may contribute to tau spread in neurodegenerative tauopathies, including AD.

There are several questions raised by experimental models that were not addressed in this study. For example, calpain-mediated cleavage of the NR2B subunit of NMDARs has been shown to give rise to active NMDAR forms that could exacerbate excitotoxicity [22, 64]. We did not observe calpain-cleaved NR2B fragments in this study, which could have been due to the effects of postmortem degradation of rapidly turned over proteins, or the levels of these fragments being below detectable levels. The transient increase in NR2B holoprotein that we observe at Braak stage II-III in supernatant fractions could imply that calpain-mediated cleavage of NR2B occurs from mid-stage AD. Alternatively, it is possible that an early compensatory response resulting in increased NR2B in Braak II-III tissues is overcome as AD develops.

In addition, we observed loss of only post-synaptic proteins in supernatants from late-stage AD cortical homogenates, with the pre-synaptic marker synapsin 1 being increased at Braak stage III and returning to control levels at end-stage AD. This result is in discrepancy to previous findings showing reductions in synapsin-1 amounts in lamina 3 of the posterior cingulate cortex in Braak stage V-VI AD brain, relative to early Braak stage and non-cognitively impaired controls [62]. However, connections from the posterior cingulate are very different to those from the temporal cortex [56], and this may account for the difference in these findings. In addition, the relatively small sample set used in this study may have masked subtle changes in protein amounts during AD progression. Furthermore, the control group used in this study included some individuals younger than average in comparison to the experimental groups. This is believed not to have skewed the findings since these samples did not appear to differ significantly from older controls. However, it would be interesting in future work to assess the contribution of normal aging to the changes described here, perhaps using resources such as that collected from the MRC-CFAS study [19] or the Lothian Birth Cohort [57].

## Conclusions

In conclusion, in this study we have used postmortem human brain to examine protein changes in different stages of AD. We provide evidence to show that alterations in calpain activity occurs relatively early in the disease process, concurrent with increased Aβ1-42 production and activation of tau kinases, and prior to increased tau phosphorylation and loss of post-synaptic markers (Fig. 6d). Our findings therefore suggest that aberrant regulation of calpain is an important early step in disease development, supporting ongoing pre-clinical and clinical studies focused on correcting disrupted calcium channel activation and calpain activation in Alzheimer’s disease and related neurodegenerative conditions. Moreover, our results suggest that there are synaptic compensatory mechanisms during early Braak stages. Further experimentation may reveal the mechanisms underlying these events and perhaps indicate strategies to prolong this supposed endogenous neuroprotective response.

### Ethics approval and consent to participate

Postmortem human brain was obtained from the MRC London Neurodegenerative Diseases Brain Bank (REC reference: 08/MRE09/38 + 5).

### Consent for publication

Not applicable.

## Additional files

Additional file 1: Table S1. Characteristics of postmortem brain samples (DOCX 75 kb) Additional file 2: Table S2. Summary of postmortem brain characteristics (DOC 28 kb)

## Abbreviations

AD: Alzheimer’s disease
Aβ: β-amyloid
AMPA: α-amino-3-hydroxy-5-methyl-4-isoxazolepropionic acid
APP: amyloid precursor protein
CAST: calpastatin
CDK5: cyclin-dependent kinase 5
GSK3: glycogen synthase kinase 3
MAPK: mitogen activated protein kinase
NFT: neurofibrillary tangle
NMDAR: N-methyl-D-aspartate receptor
NMNAT1: nicotinamide mononucleotide adenylyltransferase 1
NSE: neuron specific enolase
PSD95: postsynaptic density-95
PICALM: phosphatidylinositol binding clathrin assembly protein
SARM1: sterile alpha and TIR motif-containing protein
