# Supplementary material for: Upregulation of calpain activity precedes tau phosphorylation and loss of synaptic proteins in Alzheimer’s disease brain
Source: Acta Neuropathol Commun. 2016 Mar 31;4:34. doi: 10.1186/s40478-016-0299-2 (PMC4818436; doi:10.1186/s40478-016-0299-2)
Supplement: Additional file 1: Table S1. — Characteristics of postmortem brain samples (DOCX 75 kb) [file 40478_2016_299_MOESM1_ESM.docx]

**Supplementary Table 1: Characteristics of postmortem brain samples**

Temporal cortex was obtained from postmortem control brains (CTRL), and those with Braak stage II-VI sporadic Alzheimer’s disease (AD). Postmortem delay (PMD), age and sex were balanced between groups as far as possible. *indicates samples from which tissue was used to generate representative IHC images.

| Diagnosis | Case number | Sex | Age (years) | PMD (h) | Case notes |
| --- | --- | --- | --- | --- | --- |
| CTRL* | 1 | F | 87 | 22 | Normal adult brain |
| CTRL | 2 | M | 78 | 47 | Early tau pathology, no neuritic plaques |
| CTRL | 3 | F | 82 | 13 | Argyrophilic grains low to moderate density |
| CTRL | 4 | M | 59 | 50 | Normal adult brain |
| CTRL | 5 | M | 40 | 40 | Normal adult brain |
| II | 15 | F | 92 | 17 | Some tau deposition |
| II* | 16 | F | 90 | 50 | Mild Alzheimer’s-type changes and mild amyloid angiopathy |
| II | 17 | M | 93 | 33 | Mild Alzheimer’s-type changes |
| II | 18 | F | 84 | 35 | Alzheimer’s changes, consistent with patient’s age |
| III | 19 | M | 92 | 11 | Mild Alzheimer’s-type changes |
| III | 20 | F | 70 | 38 | Possible Alzheimer’s disease (CERAD) BNE stage III |
| III* | 21 | M | 86 | 52 | Ageing changes |
| IV | 22 | M | 82 | 28 | Alzheimer’s disease with limbic TDP-43 pathology |
| IV | 23 | M | 86 | 53 | Alzheimer’s disease with extensive severe amyloid angiopathy |
| IV | 24 | F | 83 | 22 | Alzheimer’s disease (limbic stage) and moderate to severe amyloid angiopathy |
| IV* | 25 | F | 89 | 56 | Alzheimer’s disease HP tau stage VI severely affecting limbic system and moderately extending to neocortex |
| V | 26 | F | 80 | 13 | Alzheimer’s disease with mild amyloid angiopathy |
| V | 27 | F | 82 | 69 | Alzheimer’s disease with mild amyloid angiopathy |
| V* | 28 | M | 86 | 26 | Alzheimer’s disease with moderate amyloid angiopathy |
| VI | 29 | F | 84 | 36 | Braak VI |
| VI* | 30 | M | 88 | 46 | Braak VI |
| VI | 31 | F | 92 | 42 | Braak VI |
| VI | 32 | F | 69 | 16 | Braak VI |
| VI | 34 | F | 82 | 5 | Braak stage VI, mild amyloid angiopathy |

* Indicates tissue shown in immunohistochemistry studies.

**Supplementary Table 2: Summary of postmortem brain characteristics**

Summary of the characteristics of each sample group, including sex, age and average postmortem delay (PMD). Data shown is mean and standard deviation (SD).

| **Stage** | **Gender** | **Age: mean (SD)** | **PMD: mean (SD)** |
| --- | --- | --- | --- |
| CTRL | 2F; 3M | 69.20 (19.46) | 34.4 (16.2) |
| II | 3F; 1M | 89.75 (4.03) | 33.8 (13.5) |
| III | 1F; 2M | 82.67 (11.37) | 33.7 (20.8) |
| IV | 2F; 2M | 85.00 (3.16) | 39.8 (17.3) |
| V | 3F; 1M | 82.67 (3.05) | 36.0 (29.3) |
| VI | 4F; 1M | 83.00 (8.72) | 29.0 (17.7) |
