# Supplementary material for: Upregulation of calpain activity precedes tau phosphorylation and loss of synaptic proteins in Alzheimer’s disease brain
Source: Acta Neuropathol Commun. 2016 Mar 31;4:34. doi: 10.1186/s40478-016-0299-2 (PMC4818436; doi:10.1186/s40478-016-0299-2)
Supplement: Additional file 2: Table S2. — Summary of postmortem brain characteristics (DOC 28 kb) [file 40478_2016_299_MOESM2_ESM.doc]

**Supplementary Table 2: Summary of postmortem brain characteristics**

Summary of the characteristics of each sample group, including sex, age and average postmortem delay (PMD). Data shown is mean and standard deviation (SD).

| **Stage** | **Gender** | **Age: mean (SD)** | **PMD: mean (SD)** |
| --- | --- | --- | --- |
| CTRL | 2F; 3M | 69.20 (19.46) | 34.4 (16.2) |
| II | 3F; 1M | 89.75 (4.03) | 33.8 (13.5) |
| III | 1F; 2M | 82.67 (11.37) | 33.7 (20.8) |
| IV | 2F; 2M | 85.00 (3.16) | 39.8 (17.3) |
| V | 3F; 1M | 82.67 (3.05) | 36.0 (29.3) |
| VI | 4F; 1M | 83.00 (8.72) | 29.0 (17.7) |
